# Treatment of atrial fibrillation with a dual defibrillator in heart failure patients (TRADE HF): protocol for a randomized clinical trial

**DOI:** 10.1186/1745-6215-12-44

**Published:** 2011-02-15

**Authors:** Giovanni Luca Botto, Giuseppe Boriani, Stefano Favale, Maurizio Landolina, Giulio Molon, Claudio Tondo, Mauro Biffi, Giuseppe Grandinetti, Paolo De Filippo, Giovanni Raciti, Luigi Padeletti

**Affiliations:** 1 U.O. di Cardiologia, Ospedale S. Anna, Como, Italy; 2 U.O. Cardiologia ed Ematologia, Policlinico S.Orsola Malpighi, Bologna, Italy; 3 D.E.T.O., Sez. di Cardiologia Universitaria, Osp. Policlinico Consorziale, Bari, Italy; 4 Laboratorio di Elettrofisiologia, Fondazione Policlinico San Matteo IRCCS, Pavia, Italy; 5 U. O. di Cardiologia, Ospedale Sacro Cuore, Negrar (VR), Italy; 6 Area Aritmologia, Fondazione Centro Cardiologico Monzino IRCCS, Milano, Italy; 7 U.O. Cardiologia ed Ematologia, Policlinico S.Orsola Malpighi, Bologna, Italy; 8 D.E.T.O., Sez. di Cardiologia Universitaria, Osp. Policlinico Consorziale, Bari, Italy; 9 U.O. di Cardiologia, Ospedali Riuniti, Bergamo, Italy; 10 Boston Scientific, Milano, Italy; 11 Ist. di Clinica Medica I° e Cardiologia, A.O. Di Careggi, Firenze, Italy

## Abstract

**Background:**

Heart failure(HF) and atrial fibrillation(AF) frequently coexist in the same patient and are associated with increased mortality and frequent hospitalizations. As the concomitance of AF and HF is often associated with a poor prognosis, the prompt treatment of AF in HF patients may significantly improve outcome.

**Methods/design:**

Recent implantable cardiac resynchronization (CRT) devices allow electrical therapies to treat AF automatically. TRADE-HF (trial registration: NCT00345592; http://www.clinicaltrials.gov) is a prospective, randomized, double arm study aimed at demonstrating the efficacy of an automatic, device-based therapy for treatment of atrial tachycardia and fibrillation(AT/AF) in patients indicated for CRT. The study compares automatic electrical therapy to a traditional more usual treatment of AT/AF: the goal is to demonstrate a reduction in a combined endpoint of unplanned hospitalizations for cardiac reasons, death from cardiovascular causes or permanent AF when using automatic atrial therapy as compared to the traditional approach involving hospitalization for symptoms and in-hospital treatment of AT/AF.

**Discussion:**

CRT pacemaker with the additional ability to convert AF as well as ventricular arrhythmias may play a simultaneous role in rhythm control and HF treatment. The value of the systematic implantation of CRT ICDs with the capacity to deliver atrial therapy in HF patients at risk of AF has not yet been explored. The TRADE-HF study will assess in CRT patients whether a strategy based on automatic management of atrial arrhythmias might be a valuable option to reduce the number of hospital admission and to reduce the progression the arrhythmia to a permanent form.

**Trial registration:**

NCT00345592

## Background

Heart failure (HF) and atrial fibrillation (AF) are two of the major diseases affecting older adults and, when chronic, are often associated with increased mortality, frequent hospitalizations and decreased quality of life [1-3]. These two disorders share similar characteristics and frequently coexist in the same patient, with the prevalence of AF increasing together with the severity of HF [4-7]. The association of AF with a worse prognosis in HF patients is still under debate with controversial results in favor [2,5,8] or against [9,10]. Even though data regarding improved survival rates is currently lacking, as far as health care is concerned, one goal of AF treatment in HF patients is to reduce hospitalization and improve quality of life [11]. The recent introduction of an implantable device which can provide both cardiac resynchronization therapy (CRT) and ventricular and atrial electrical therapies, paves the way toward the combined treatment of both diseases. In fact, CRT has proven to be effective in correcting asynchrony in HF [12-14], while defibrillation therapy has demonstrated its effectiveness in treating both ventricular and atrial arrhythmias [15-17]. Due to the high prevalence of AF in HF patients who meet indications for an implantable cardioverter defibrillator (ICD) [18,19], this combined therapy could treat HF, prevent sudden cardiac death, prevent AF and restore sinus rhythm when necessary. As AF may compromise the efficacy of CRT in terms of reverse remodeling [20,21] a break in the AF/HF cycle could improve prognosis both by optimizing HF treatment and restoring sinus rhythm. The TRADE-HF study has been designed to assess the benefits of an automatic, device-based management of atrial arrhythmias in patients suffering from symptomatic HF, indicated for CRT treatment with defibrillation backup and implanted with a dual (atrial and ventricular) defibrillator.

## Methods/Design

### Study Design and Objectives

The TRADE-HF is a prospective, open-label, randomized, multi-center, two parallel arm study. The study has been approved by the local Ethic Committees of all 20 participating centers and has been registered to http://www.clinicaltrials.gov site (NCT00345592). Enrollment started in November 2006. The steering committee of the study is responsible for the study design and its amendments.

**Figure 1:**
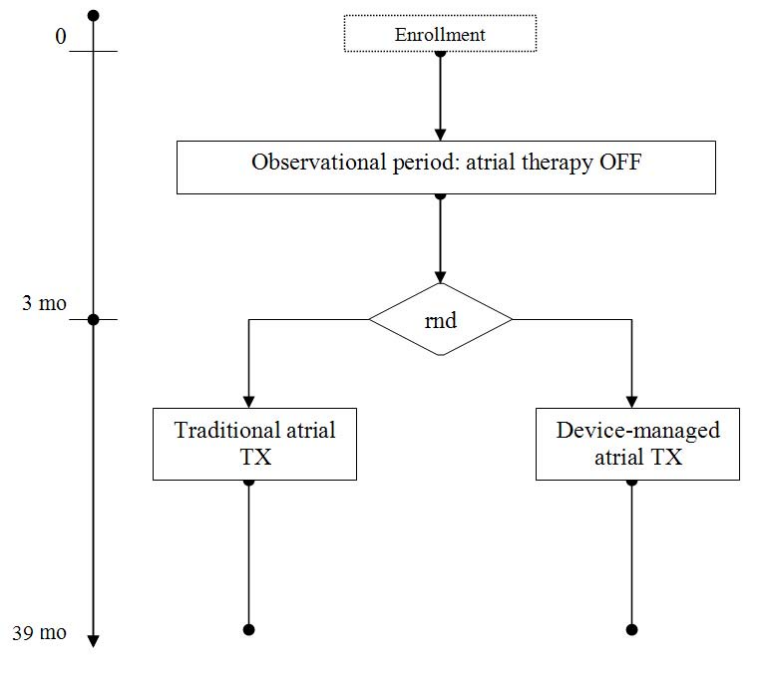
**Study flowchart**. RND: Randomization.

Primary objective of the TRADE-HF study is to demonstrate a reduction in the combination of hospitalizations for cardiovascular causes or death for cardiovascular causes or development of permanent AF, using a device-based treatment of atrial arrhythmias (i.e. automatic therapy) compared to a traditional approach including hospitalization for symptoms and and/or in-hospital treatment of AT/AF (i.e. traditional therapy). Automatic therapy implies that atrial arrhythmias are detected by the device itself which treats the rhythm automatically within 3 hours after episode onset with a predetermined sequence of programmed electrical therapies (anti-tachycardia pacing and electrical shock). On the other side in the traditional arm automatic shock therapy is not active; therefore, patients who will experience symptomatic atrial arrhythmias at home will refer to their center, where treatment of the atrial arrhythmia will be done according to center's practice (e.g. drugs, external cardioversion, device shock commanded by the physician in a hospital environment). Hospitalizations for cardiovascular causes will include any unplanned cardiac cause hospitalization including day hospital, access to the emergency units and in-hospital procedures of AT/AF cardioversion. Permanent AF is considered as any form of AF that is found to be refractory to drug or electrical therapy, for which the physician resigns to take additional actions to restore sinus rhythm and that persists additionally for at least one month. After obtaining informed consent, a three-month observation period ensues, during which the device parameters are optimized, data on atrial arrhythmias are collected and patient's response to the device treatment is assessed. Thereafter, patients are randomized to the two different treatment arms, automatic and traditional therapy in a 1:1 ratio. After being randomized, patients are followed for three years to assess endpoints. The study flowchart is shown in figure 1.

Secondary objectives include: the performance assessment of automatic atrial therapies, and a comparison between the automatic-managed and traditional therapy arm of the following outcomes: in-hospital costs, Quality of Life (QoL) scores, number of hospital admissions and the separate components of the primary endpoint.

An exploratory analysis will be done in order to investigate the response to CRT treatment for the enrolled patients. A positive response to CRT is considered a relative LVEF increase ≥ 20% and/or a relative LVESV decrease ≤ 15% measured at echocardiographic recording associated with a functional assessment defined as reduction of at least one NYHA class or of 15 points at Minnesota living with heart failure score for NYHA class III patients or at least stable NYHA class or 10 points decrease at Minnesota living with heart failure score for NYHA class II patients. This definition reflects patient's cardiac remodeling associated to a functional improvement in highly symptomatic patients or at least a stable functional condition in mild symptomatic patients. The proportion of CRT responders will be analyzed at time of randomization and one year after randomization; additionally a correlation, separately at the two time points, will be searched among the proportion of CRT responders, the burden of atrial fibrillation (total percentage of recorded AT/AF episodes: 0; <10%; >10%) and the target site of left ventricular lead (lateral wall vs. other).

### Patient Selection

Patients who meet all of the following criteria are considered eligible to take part in the study: 1) chronic (>6 weeks) symptomatic HF despite stable, optimal drug therapy for HF and AF, 2) patients scheduled for and implanted with a cardiac resynchronization device with defibrillator backup according to current guidelines. 3) age 18 or above, or of legal age to give informed consent according to specific national regulations. Principal exclusion criteria include: 1) chronic or persistent atrial fibrillation refractory to medical or electrical therapy 2) valve disease, and/or patients with or who are likely to receive a mechanical tricuspid valve during the course of the study 3) patients who have undergone (or are scheduled to undergo) an intervention of atrial fibrillation ablation 4) cerebral vascular event or transient ischemic attack within 12 months of implantation which lead to significant impairment 5) life expectancy of less than 1 year due to other medical conditions 6) NYHA class IV. According to the listed criteria, patients are not selected on the basis of atrial arrhythmia recurrence, except for chronic/persistent AF.

### Interventions and Follow Up

After obtaining informed consent, patients' baseline data are collected, including demographic background and medical history. A clinical evaluation performed at the time of enrollment includes NYHA class assessment, an electrocardiogram, an echocardiogram and Qol assessment using the Minnesota-Living With Heart Failure questionnaire. During the three-month observation period before randomization, we collect data on the incidence of supraventricular arrhythmia and clinical events; deaths from any cause or chronic atrial fibrillation before randomization are considered dropouts. Patients are randomized by means of a centralized, computer-generated randomization list, with a 1:1 ratio; given that both patients and physicians are aware of the randomized treatment and that patients are specifically trained to manage their symptoms in two different ways depending on the study arm, patients and investigators are not blinded with respect to treatment. At the randomization visit, NYHA class, QoL and atrial and ventricular function are assessed (the latter by echocardiography) in order to evaluate the response to CRT. After randomization, patients are followed up for a total duration of 3 years, with planned ambulatory visits every 6 months.

At follow-up visits a clinical assessment is performed including NYHA class staging, ECG and medical therapy. QoL assessment and echocardiography are mandatory one and two years after randomization. Data regarding events such as hospitalization are collected over the entire follow-up period.

Device shocks and all interventions performed to treat atrial arrhythmias (drug, electrical or other therapy, repeated or not) are collected for both study arms. A protocol for the management of atrial arrhythmias in the traditional arm has not been predefined, but left to center's specific therapeutic practice and decision making, and may range from simple observation of the response to drugs of the hospitalized patient to repeated attempts of internal cardioversion done with the device in a in-hospital environment. The only requirements for this arm are 1) automatic shock set to "off" and 2) arrhythmia treatment performed at in-hospital environment.

If a primary endpoint event, other than patient death, occurs, the patient continues to be followed over the entire 2-year period in order to assess secondary endpoints. Crossover may occur at any point during the follow-up: patients may withdraw from the device-controlled therapy arm when atrial shock therapy is disabled due to severe discomfort related to shocks or too frequent atrial shock therapies; patients may cross from the traditional therapy to the device-controlled arm when atrial shock therapy is enabled chiefly because the number of hospital admissions for atrial cardioversion becomes too frequent. Patients who present with permanent/chronic AF, regardless of the randomization arm, start rate control therapy with the device's atrial therapy set to off: this is not considered a crossover, as the patient has already reached the primary endpoint.

### Sample Size

Patients treated for CRT in two large randomized clinical trials, the COMPANION [13] and CARE-HF [14] studies, yielded a respective incidence of 62% and 38% in deaths or hospitalizations due to cardiac reasons after a two-year follow-up period. According to the AFFIRM study [22], the evolution to chronic AF may range between 20% and 25% after two years, in patients who are likely to experience recurrent AT/AF. In HF patients, the prevalence of AF is reported to vary from 10% to 50%, with higher values in older patients and in those with superior NYHA class [23]. Additionally, the incidence of new-onset AF in these patients ranges from 3% and 10% per year [23]. A study of a large cohort of heart failure patients has recently revealed that around one third of hospitalized patients had ECG evidence of AF [24]. Based on these data, we assumed that around 35% of patients in both study arms of the TRADE-HF study will have experienced at least one episode of atrial arrhythmia after three years and that around 60% will have experienced at least one hospitalization for cardiac reasons or persistent/chronic AF or death from cardiovascular causes. Patients treated automatically with the device are expected to have a lower rate of events. The effectiveness of treating AT/AF with a dual-defibrillator is very high and reported to be around 90% in different settings [15,16]. Assuming a 60% risk for combined endpoints in the traditional arm versus a 43% risk in the device-managed arm, using a two-tailed alpha of 0.05, with a Cox proportional hazards regression model the study will need a total of 360 patients (180 per group) in order to achieve a power of 90% and demonstrate a difference in primary endpoint rates among patients in the device-managed arm versus those in the traditional arm. Assuming a 15% attrition rate during the study, due to loss to follow-up or failure to adhere to the study design, the total sample size will amount to 414 patients (207 per group).

### Data Analysis

Descriptive statistics will be presented for the collected variables, overall and according to the randomization group; for categorical variables absolute and relative frequencies; for continuous variables mean, standard deviation, median, 25th-75th percentile, minimum and maximum. Baseline characteristics of the two study arms will be listed. Cox modeling will be used to perform the primary analysis and assess the association between treatment (traditional vs. device-managed) and the primary endpoint. Hazard ratios and their 95% confidence intervals will be presented together with Kaplan-Meier cumulative survival and with event rates (per 100 persons per year) in each group. The model will include the following potential confounders: evidence of AT/AF in the patient's medical history or recorded by the device before randomization and patients' response to CRT at randomization. In particular, group effects will be tested by interaction terms comparing the treatment effect in patients who are or are not responders to CRT at randomization. Competing events (causes of death other than cardiovascular, transplant or ventricular assist device implant) will be censored at the time they occur. The primary analysis will be done according to Intention to treat (ITT) i.e. automatic shocks programmed according to randomization. Survival analysis techniques will be used to evaluate time-to-event for the evolution to chronic AF and for both subgroup analyses (CRT responders and AT/AF). In case the primary endpoint is fulfilled, additional post-hoc analyses will be performed. In particular a positive response to CRT responsiveness will be analyzed with respect to the planned variables by means of a logistic regression model. The device's ability to manage and correctly classify atrial arrhythmia will be evaluated considering the sensitivity, specificity and accuracy of the device's classification compared to that made by the Event Committee. Quality of life scores, number and total duration of hospital admissions (for all causes, cardiac causes and for heart failure), total duration of AF and total costs will be compared between the two study arms after 3 years using the appropriate statistical test, according to distribution. For economic analysis costs are estimated for hospitalization (reasons, entry date, duration, department), interventions and instrumental examinations performed. A P value of at least 0.05 will be used for statistical significance.

### Data Safety and Monitoring Plan

Prior to initiating the study at each site, the Sponsor shall visit the site to ensure understanding of the protocol and that the involved staff has sufficient time and facilities to conduct the study. During the course of the study additional visits may be conducted at regular intervals, to assess the continued compliance with the study protocol and applicable regulations, that data are collected in a timely accurate and complete manner and that study records are adequately maintained at the site. Necessity and frequency of site visits are determined on basis of the data quality and completeness, as periodically retrieved with the interrogation of the online database of the study. All Study Events, including Adverse Events (whether serious or not), patient withdrawals, deviations and therapies to treat AF, are reported to the Sponsor via electronic e-mail notification and processed within one working day. Adverse Events are analyzed, and reported to competent authorities when needed. Investigators are responsible for providing the Sponsor with a description of each reported adverse event including the suspected cause, what corrective actions were taken and what the clinical outcome was for the patient. All events relevant for primary endpoint analysis, including recorded episodes of atrial arrhythmias will be adjudicated by a board of independent physicians (Event Committee) blinded with respect to device classification and to the randomization arm.

## Discussion

Both heart failure and atrial fibrillation affect several million patients in the United States and cause substantial morbidity and mortality [1-3] representing a serious issue in terms of health care. The link between the two pathologies is well-documented: the presence of HF has been identified as one of the most powerful independent predictors of AF, with a 6-fold increase in the relative risk of its development [4], while the prevalence of AF increases together with the severity of HF and ranges from 5% in NYHA classes I-II to 45% in NYHA class IV [5,6], with a higher incidence of new onset AF in patients with more severe HF [7]. Additionally, the two diseases share common risk factors such as hypertension, diabetes, coronary artery disease and valve disease [2]. As far as prognosis is concerned, AF as a co-morbidity affects both morbidity and mortality: patients affected by both diseases have an increased risk of hospitalization and embolic events, with AF being a non-independent predictor of mortality [5]. This association between HF and the development of AF has been emphasized as a two-fold vicious cycle, and the issue as to whether an effective treatment of one of the two conditions may have an effect on the other has recently become a subject of interest [11,25]. AF treatment and sinus rhythm maintenance, (i.e. rhythm control) is considered to have several advantages in HF patients such as improved hemodynamic, restoration of contractile function and the possible regression of tachycardiomyopathies. Analyses conducted on the AFFIRM and DIAMOND data revealed favorable outcomes of rhythm control in HF subsets [26,27]. Nevertheless, data from a large prospective randomized study, the AF-CHF trial [28,29] enrolling patients with a left ventricular ejection fraction of 35% or less, symptoms of congestive heart failure, and a history of atrial fibrillation, assessed that a routine strategy of rhythm control does not reduce the rate of death from cardiovascular causes, as compared with a rate-control strategy. On the other hand, patients with cardiac dyssynchrony and reduced ejection fraction, currently meet class I indications for CRT. Additionally, recent guidelines have introduced this option also for patients with persistent AF and indications for AV junction ablation (class IIa, level of evidence C) [30]. As only few patients enrolled in the AF-CHF trial were meeting indications for ICD or CRT (only 7% had an implantable device according to current international practice) the concomitant treatment of HF and AF through resynchronization and automatic cardioversion in this patient may lead to a different picture. A particular care should be deserved for those HF patients with low burden of AF or who did not show clinical episodes of AF at the time of device implant, and that may develop AF over time, that are reported to reach up to 20%-25% of patients in a mid-term timeframe [20,31]. Indeed some studies showed that new onset AF after CRT implantation is associated with a poor response to CRT, [20,21] and that a positive response to CRT, on the other side, is associated with a decreased AF burden [32]: those subjects may therefore benefit from the concomitant treatment of HF through resynchronization and AF though rhythm control.

In this panorama, developments in implantable devices pave the way toward the treatment of the two concomitant diseases by a single device: a CRT pacemaker with the additional ability to convert AF as well as ventricular arrhythmias may play a simultaneous role in rhythm control and HF treatment. The safety and performance of internal atrial cardioversion has been already tested in ICD recipients without heart failure. Internal atrial cardioversion has been shown to be an effective treatment for atrial arrhythmias ensuring the successful conversion of most AF episodes, often requiring only a single shock or atrial anti-tachycardia pacing (ATP) and leading to a decrease in the frequency of long-lasting episodes [16-18]. However, the benefits of dual chamber ICDs equipped with atrial therapies go beyond the efficacy of atrial shocks as this therapy could be able to: relieve patients' symptoms, limit adverse events such as stroke, thromboembolism, recurrent hospitalizations, improve hemodynamic function and prevent ventricular tachycardia or fibrillation triggered by atrial tachyarrhythmias [17,33]. As regards heart failure, the value of the systematic implantation of CRT ICDs with the capacity to deliver atrial therapy in HF patients with a history (or at risk) of AF, has not yet been explored and some authors have suggested that this aspect be examined in prospective studies [11,34,35]. Implantable CRT devices that include low energy atrial cardioversion as well as electrical therapy for ventricular arrhythmias, have recently made it possible to explore this field. The TRADE-HF study is designed to assess whether a strategy by means of automatic management of atrial arrhythmias might be a valuable option to reduce the number of cardiovascular cause hospital admission, death for cardiovascular causes and the progression the arrhythmia to a persistent form. After receiving thorough training on enrollment, it is assumed that patients without the automatic atrial shock will refer to a hospital if severe or persisting symptoms occur, which could lead to AF diagnosis and cardioversion if required. Therefore, AF will be adequately treated in both arms, albeit diagnosis, timing and methods of treating the arrhythmia will vary. Although we do not know whether these different methods for delivering atrial therapy may have a different impact specifically on quality of life and the number of hospital admissions for heart failure, the automatic therapy arm would appear to have several advantages. Automatic atrial shocks, when therapy is successful and in the absence of recurrences, successfully manages electrical cardioversion without the need for hospital treatment, thus reducing the number of hospital admissions. Automatic therapy may shorten the time between AF onset and sinus rhythm restoration when compared to the in-hospital approach. One possible (positive) side effect of this timely approach is a reduced occurrence of successive atrial arrhythmias as well as possible fewer hospitalizations for heart failure due to the detrimental effect of AF. Finally, automatic therapy can treat asymptomatic episodes promptly: with a traditional approach based on symptoms and in-hospital cardioversion, these episodes may not be detected until they degenerate into symptomatic HF. In general, the automatic treatment of AF may be an option for a prompt treatment strategy, while a more traditional approach, based on in-hospital cardioversion, may delay effective treatment, and possibly worsen heart failure. Despite this study is not powered to explore this potential specific clinical benefit, any differences observed in the component of the primary endpoints or in secondary endpoints (i.e permanent AF or cardiac cause hospitalizations) could be useful to generate further hypotheses on this topic.

Early treatment of AF also seems to be justified as far as histopathology is concerned, as arrhythmia is not only an electrical pathology, but a condition that, over an extended time, may induce non-reversible organic diseases such as fibrosis and conduction abnormalities [34-37]. In light of this, early AF treatment could be reserved even for those patients with a lower AF burden or for those with no history of AF. A recent work by Saxon et al. reports that 25% of CRT candidates with a history of AF, experience recurrent AF within 6 months of implant [38]. Given the high prevalence of AF in patients currently indicated to CRT, and the opportunity to treat new onset AF promptly, the inclusion criteria of the TRADE HF study consider all patients implanted with a biventricular device with defibrillator, regardless of their atrial arrhythmic history.

Finally, whether CRT may have an effect on AF burden in patients with persistent or paroxysmal AF is still a matter of research and prospective studies to evaluate this impact have been encouraged [33]. It is not known whether reverse remodeling, induced by biventricular pacing in patients who respond to CRT, may have an effect on the incidence of AT/AF: minimal data exist on the effect of long term CRT on left atrial dimensions and/or LV reverse remodelling in patients with AF and the results are contradictory [8,31,39] even if a recent. The TRADE HF study will analyze if there are substantial differences in the incidence and characteristics of atrial fibrillation between CRT responders and non-responders.

## Competing interests

Giovanni Raciti is employed at Boston Scientific.

## Authors' contributions

The steering committee of the TRADE-HF study (GLB, GB, SF, ML, GM, CT ) contributed to the study design, all the other authors were involved in the drafting and revision of the manuscript.

### Appendix - List of investigators and participating centers

M.Anaclerio, G.Luzzi, A.Scialpi, D.E.T.O., Sez. di Cardiologia Universitaria, Osp. Policlinico Consorziale, Bari; F. Cantù, R.Brambilla, U.O. di Cardiologia, Ospedali Riuniti, Bergamo; M.Bertini, L.Frabetti, C.Martignani, C.Valzania, Ist. di Cardiologia, Università di Bologna, A.O. S.Orsola Malpighi, Bologna; G. Giannola, R.Torcivia, U.O. di Cardiologia, Osp Civico G,Giglio, Cefalù (PA); G. Senatore, M.Giuggia, G.Trapani, U.O. di Cardiologia, P.O. Riunito, Ciriè (TO); M.Luzi, G.Russo, B.Mariconti, U.O. di Cardiologia, Struttura di Elettrofisiologia,Ospedale S. Anna, Como; G. De Rinaldis, M. Galluccio Mezio, M. Galluccio Mezio, U.O. di Cardiologia, P.O. San Giuseppe da Copertino, Copertino (LE); G.Ricciardi, N.Musilli, P.Pieragnoli, Laboratorio di Elettrofisiologia, Ist. di Clinica Medica I° e Cardiologia, A.O. Di Careggi, Firenze; O. Bramanti, F.Lucà, C.Melluso, G.Piccolo, U.O. di Cardiologia, Az.Osp.Universitaria Policlinico "G.Martino" Messina; P.Belli, G.Covino, P.Capogrosso, U.O. di Cardiologia, Osp. S. Giovanni Bosco, Napoli; E.Barbieri, A.Costa, M.Corso, U. O. di Cardiologia, Servizio di Elettrofisiologia, Ospedale Sacro Cuore, Negrar (VR); O. Pensabene, F.Ingrillì, U.O. di Cardiologia, A.O. Villa Sofia CTO, Palermo; A.Vicentini, R.Rordorf, B.Petracci, S.Savastano, Laboratorio di Elettrofisiologia, Fondazione Policlinico San Matteo IRCCS, Pavia; M. Piacenti, L.Panchetti, A.Rossi, U.Startari, Area di Ricerca San Cataldo, U.O. di Cardiologia CNR - Istituto Fisiologia Clinica, Pisa; F. Doni, A.Kheir, M.Manfredi, U.O. di Cardiologia, Policlinico S. Pietro, Ponte S. Pietro (BG); M. Liccardo, P.Nocerino, G.Sibilio, U.O. di Cardiologia, O. Civile S.Maria delle Grazie, Pozzuoli (NA); T. Giovannini, F.Frascarelli, U.O. di Cardiologia, P.O. Misericordia e Dolce, Prato.; G. Speca, A. Poggi, Centro di Cardiologia e Cardiostimolazione, P.O. G.Mazzini, Teramo; R. Massa, G.Trevi, C.Amellone, P.Golzio, Cardiologia 1, A.O. San Giovanni Battista Molinette, Torino; S. Marra, M.Sicuro, C.Budano, Cardiologia 2, A.O. San Giovanni Battista Molinette, Torino.
